# The Prevalence and Characteristics of Mitral Regurgitation in Heart Failure: A Chart Review Study

**DOI:** 10.31083/j.rcm2307235

**Published:** 2022-06-24

**Authors:** Chengchen Zhao, Chunna Jin, Yimin Shen, Xiaoping Lin, Yi Yu, Meixiang Xiang

**Affiliations:** ^1^Department of Cardiology, The Second Affiliated Hospital, Zhejiang University School of Medicine, Key Lab of Cardiovascular Disease of Zhejiang Province, 310009 Hangzhou, Zhejiang, China

**Keywords:** functional mitral regurgitation, heart failure, prevalence, associated factors

## Abstract

**Background:**

Mitral regurgitation (MR) is one of the common complications of heart failure (HF). The prevalence and characteristics of MR are rarely investigated, especially in the Chinese population.

**Objectives:**

The purpose of this study was to determine the prevalence and characteristics of non-organic MR in HF patients and subgroups defined by ejection fraction.

**Methods:**

A single-center, hospital-based, and retrospective chart review study included patients with heart failure admitted to the cardiovascular department from January 2017 to April 2020. Demographic characteristics, laboratory results, and echocardiogram results before discharge were analyzed in different groups defined by left ventricular ejection fraction (EF) using logistic regression and adjusted for confounders.

**Results:**

Finally, 2418 validated HF patients (age 67.2 ± 13.5 years; 68.03% men) were included. The prevalence of MR was 32.7% in HF, 16.7% in HF with preserve EF patients, 28.4% in HF with mid-range EF patients and 49.7% in HF with reduced EF (HFrEF) patients. In the HF with preserved EF group, multivariable logistic regression showed that 4 factors associated with MR including EF (odds ratio (OR) 0.954 (0.928–0.981), *p *= 0.001), left ventricular posterior wall thickness in diastolic phase (LVPWd) (OR 0.274 (0.081–0.932), *p *= 0.038), left atrium (LA) dimension (OR 2.049 (1.631–2.576), *p <* 0.001) and age (OR 1.024 (1.007–1.041), *p *= 0.007). In the HF with midrange EF group, multivariable logistic regression showed that 3 factors associated with MR including LA dimension (OR 2.009 (1.427–2.829), *p <* 0.001), triglycerides (TG) (OR 0.552 (0.359–0.849), *p *= 0.007) and digoxin (OR 2.836 (1.624–4.951), *p <* 0.001). In the HFrEF group, multivariable logistic regression showed that 7 factors associated with MR including EF (OR 0.969 (0.949–0.990), *p *= 0.004), (OR 0.161 (0.067–0.387), *p <* 0.001), LA dimension (OR 2.289 (1.821–2.878), *p <* 0.001), age (OR 1.016 (1.004–1.027)), *p *= 0.009), TG (OR 0.746 (0.595–0.936), *p *= 0.011), diuretics (OR 0.559 (0.334–0.934), *p *= 0.026) and ICD (OR 1.898 (1.074–3.354), *p *= 0.027).

**Conclusions:**

HF patients had a high burden of MR, particularly in the HFrEF group. Worsen cardiac structure (LA dimension and LVPWd) and function (EF), age, and medical treatment strategy played essential roles in MR.

## 1. Introduction

Heart failure (HF) remains a critical condition with a high global burden and poor prognosis [1, 2]. Mitral regurgitation (MR) following HF is distinguished by a structurally normal mitral valve and apparatus, which is frequently referred to as functional MR [3]. The prevalence of MR in HF patients ranges from 6.1% to 32.7% [4, 5]. It is associated with poor prognosis, high mortality rate, and worse life quality in HF patients [4, 5, 6, 7, 8, 9, 10].

Through the MitraClip system (Abbott Structural Heart), MR seemed to be a promising therapeutic target in patients with HF; however, two randomized clinical trials yielded opposite result on the major endpoint (hospitalization for heart failure or all-cause mortality) [11, 12]. The divergent inclusion criteria could have been attributed to the controversial results, and it raised many concerns [13]. Therefore, identifying the characteristics of MR is of growing importance.

The mechanisms underlying MR in heart failure include displacement of papillary muscles, tethering of chordae tendinae and leaflets, and annular dilation. Hence, MR is classified into the ventricle and the atrial types [3, 14]. Previous studies reported that sex, race, age, and dyslipidemia are associated with poor prognosis of MR [4, 6]. Population-based studies in HF, on the other hand, provided less clinical information concerning MR [1, 2, 15]. Hospital-based studies had reported the prevalence of MR in patients with HF, but these studies were limited to the specific types of HF and geographic regions; additionally, the clinical characteristics of MR in heart failure patients remained to be studied [4, 5, 6, 7, 8, 9, 10].

The purpose of this study was to determine the prevalence and characteristics of non-organic MR in HF patients and subgroups defined by ejection fraction. We identified the clinical characteristics of MR in a Chinese hospital based on these findings.

## 2. Method

### 2.1 Study Design and Clinical Setting

The Second Affiliated Hospital of Zhejiang University, Institutional Review Board approved this study and granted a waiver of informed consent. This study complied with the Strengthening the Reporting of Observational Studies in Epidemiology (STROBE) reporting guideline.

It was a single-center, cross-sectional study based on the medical records in the electronic health record system. It included HF patients due to ischemic and non-ischemic etiology; the admission date was limited between January 2017 and April 2020. According to the ESC guideline, we selected and classified patients with heart failure based on symptoms description (dyspnoea, chest pain, palpitations, syncope, and edema) and test results [16].

(1) Heart failure with preserved ejection fraction (HFpEF):

LVEF ≥50%, symptoms ± signs, elevated levels of natriuretic peptides, relevant structural heart disease, and/or diastolic dysfunction.

(2) Heart failure with mid-range ejection fraction (HFmrEF):

LVEF 41%–49%, symptoms ± signs, elevated levels of natriuretic peptides, relevant structural heart disease and/or diastolic dysfunction.

(3) Heart failure with reduced ejection fraction (HFrEF):

LVEF ≤40%, symptoms ± signs.

We excluded organic valvular heart disease, apparent degenerative valve disease, congenital heart disease, hypertrophic cardiomyopathy, endocarditis, infiltrated cardiomyopathy, and pericardial disease. Patients without transthoracic echocardiography were also excluded. We classified regurgitation status using echocardiographic database records by the guideline [17]: patient with moderate or severe MR was defined as the MR group; patient with none or mild MR was defined as the non-MR group.

### 2.2 Data Collection 

The clinical database was constructed based on the Electronic Medical Record System of the Second Affiliated Hospital of Zhejiang University. Generally, all admitted patients’ records (n = 27833) in the database were screened for detection. Probable HF hospitalizations (n = 4561) were eligible for inclusion. We excluded 773 patients who met the above-mentioned exclusion criteria, 900 patients who lacked echocardiogram data, 108 patients who lacked left atrium assessment data, and 162 patients who lacked NT-proBNP or BNP data. Finally, 2418 validated HF records from January 2017 to April 2020 were included. We did not use value imputation to fill in missing values. Two clinicians randomly selected twenty records for accuracy testing.

The most recent medical record was used as the baseline for patients with multiple admission records. Clinical data were extracted on patients’ clinical status, comorbidities, medication, intervention, laboratory results, and echocardiogram results prior to discharge when patients were hemodynamically stable. The etiology of HF was classified as either ischemic or non-ischemic. Thyroid disease was defined as thyroid disease diagnosed by a physician.

A standard echocardiogram was performed at the hospital before discharge following the American Society of Echocardiography guidelines [18]. MR was measured semi-quantitatively using color Doppler to assess regurgitation fraction (the regurgitant jet area/the left atrium area) in the two-and four-chamber views at end-systole. The severity of MR was classified as mild (occupied <20%), moderate (20% < occupied < 40%), and severe (occupied ≥40%). Echocardiogram results were obtained from the database which was declared before [19], including left ventricular ejection fraction (LVEF), interventricular septum thickness in diastolic phase (IVSd), left atrium (LA) dimension (defined as the diameter of left atrium in parasternal long-axis view), left ventricular end-diastolic volume, left ventricular internal diameter in diastolic phase (LVIDd), left ventricular posterior wall thickness in diastolic phase (LVPWd), mitral and tricuspid regurgitation severity. The other echocardiogram parameters were excluded due to the partially missing data.

Laboratory results were defined as the initial assessment of hospital visits. Items were listed below: brain natriuretic peptide (BNP), N-terminal pro-B type natriuretic peptide (NT-proBNP), C-reactive protein (CRP), creatine (Cr), hemoglobin (Hb), alanine aminotransferase (ALT), glycated hemoglobin (HbA1c), free fatty acid (FFA), β-hydroxybutyrate (BHB), serum glucose (Glu), triglycerides (TG), high-density lipoprotein (HDL), and low-density lipoprotein (LDL).

### 2.3 Statistical Analysis

Continuous variables in both the MR group and the non-MR group were tested for the normal distribution (Kolmogorov–Smirnov one-sample test, all *p *≤ 0.05). Categorical and consecutive data were presented as number (%), mean ± standard deviation (if data fitted normal distribution), or median ± quartile (if data did not fit normal distribution). The *$x^{2}$* test or Fisher exact test was used for categorical variables. The unpaired *t*-test or Wilcoxon signed-rank testwas used for the comparison of consecutive variables. Univariate binary logistic regression analysis was applied to assess factors associated with MR. Stepwise multivariable logistic regression was applied in univariate analysis. Additionally, we excluded BNP and NT-proBNP from the logistic model due to missing values. Finally, we conducted a subgroup analysis to test these factors association of MR in each group (HFpEF, HFmrEF, HFrEF). Two adjusted models were adopted: model 1 included the significant covariates from the bivariate analysis; model 2 tested the same covariates as model 1, further adjusting for sex, history of coronary artery disease, and diabetes. The SPSS version 20.0 (IBM, Chicago, IL, USA) and R package version 4.1.1 (R Core Team 2020) were used for all statistical analysis with two-tailed *p* values of 0.05.

## 3. Result

### 3.1 Prevalence of MR

**Fig. 1.**
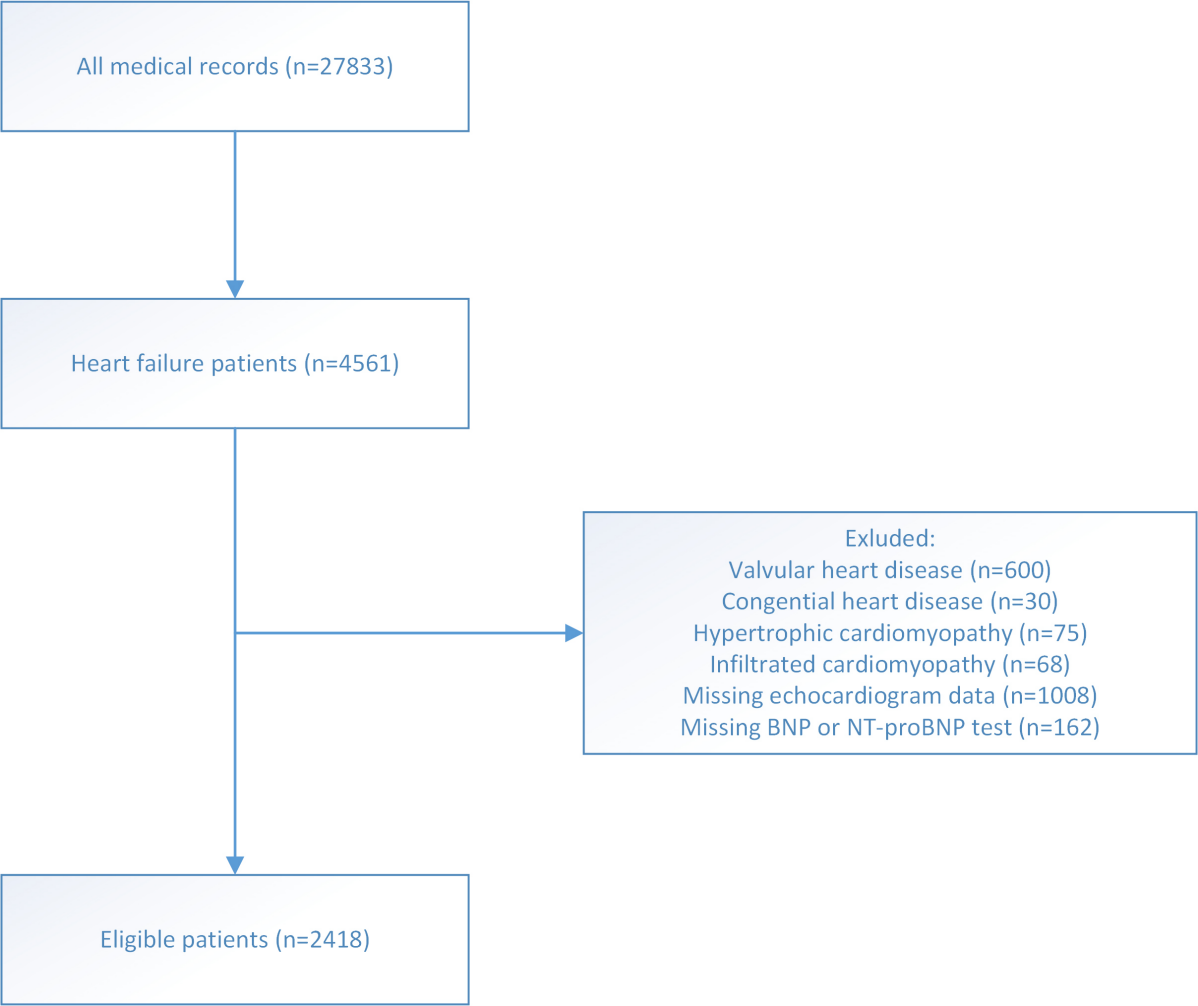
**Flow chart of patients selection**.

Overall, a total of 2418 patients were eligible for analysis (age 67.2 ± 13.5 years; 68.03% men) after selection (Fig. 1); among them, 32.71% (n = 791) had MR. 41.56% (n = 1005) had LVEF ≥50%, 15.43% (n = 373) had LVEF between 41% and 49%, 43.01% (n = 1040) had LVEF ≤40%. 16.7% (n = 168) of HFpEF group had MR, 28.4% (n = 106) of HFmrEF group had MR, and 49.7% (n = 517) of HFrEF group had MR (Table 1).

**Table 1.** **Baseline characteristics of HF subtypes**.

		HFpEF	HFmEF	HFrEF	*p*
		(n = 1005)	(n = 373)	(n = 1040)	
Clinical status					
	Mitral regurgitation (n)	168 (16.7%)	106 (28.4%)	517 (49.7%)	<0.001
	Age (y)	70.3 (12.6)	66.7 (13.7)	64.3 (13.6)	<0.001
	Sex (male)	616 (61.3%)	274 (73.5%)	755 (72.6%)	<0.001
	Body mass index (kg/$m^{2}$)	24.5 (3.68)	24.1 (3.76)	23.5 (3.82)	<0.001
	Hypertension (n)	679 (67.6%)	225 (60.3%)	477 (45.9%)	<0.001
	Diabetes (n)	275 (27.4%)	98 (26.3%)	283 (27.2%)	0.919
	Atrial fibrillation (n)	363 (36.1%)	151 (40.5%)	323 (31.1%)	0.002
	Stroke (n)	129 (12.8%)	53 (14.2%)	111 (10.7%)	0.132
	Chronic obstructive pulmonary disease (n)	84 (8.36%)	21 (5.63%)	73 (7.02%)	0.194
	Thyroid disease (n)	8 (0.80%)	3 (0.80%)	17 (1.63%)	0.202
	Chronic kidney disease (n)	105 (10.4%)	55 (14.7%)	157 (15.1%)	0.005
Heart failure cause					
	Ischemic (n)	674 (67.1%)	211 (56.6%)	468 (45.0%)	<0.001
	Non-Ischemic (n)	331 (32.9%)	162 (43.4%)	572 (55%)	<0.001

All values are presented as the means ± SD or n (%) or as the median (interquartile range). n, number of individuals.

### 3.2 Demographics and Characteristics of MR

Table 2 and Table 3 summarize the baseline characteristics of the MR and non-MR groups. Patients in the MR group and the non-MR group were comparable in terms of age and sex. Patients in the MR group had a lower body mass index (BMI), systolic blood pressure (SBP), diastolic blood pressure (DBP). Patients in the MR group had a lower rate of hypertension (MR 50.3% vs. non-MR 60.4%, *p <* 0.001) and coronary artery disease (CAD) (MR 45.0% vs. non-MR 61.3%, *p <* 0.001) (Table 2). Echocardiography parameters were analyzed. Patients in the MR group had lower EF, LVPWd, and a higher LA diameter, LVIDd (Table 2). Notably, the MR group had a significantly increased moderate to severe tricuspid regurgitation (MR 46.8.0% vs. non-MR 14.8%,* p <* 0.001). Laboratory results were displayed in Table 3.

**Table 2.** **Baseline demographic and echocardiogram characteristic of MR vs. non-MR**.

		non-MR	MR	*p*
		(n = 1627)	(n = 791)	
Clinical status age (y)		68.0 (59.0–76.0)	69.0 (59.0–78.0)	0.051
	Sex (male)	1120 (68.8%)	525 (66.4%)	0.240
	Body mass index (kg/$m^{2}$)	24.2 (22.0–26.6)	23.0 (20.8–25.5)	<0.001
	Systolic blood pressure (mmHg)	118 (105–132)	113 (101–127)	<0.001
	Diastolic blood pressure (mmHg)	68.0 (60.0–76.0)	67.0 (59.0–75.0)	0.047
	Heart rate (beat/min)	72.0 (63.0–81.0)	74.0 (65.0–83.0)	0.011
	Heart failure classification			<0.001
	Preserved	837 (51.4%)	168 (21.2%)	
	Mid-range	267 (16.4%)	106 (13.4%)	
	reduced	523 (32.1%)	517 (65.4%)	
Comorbidities				
	Hypertension (n)	983 (60.4%)	398 (50.3%)	<0.001
	Coronary artery disease (n)	997 (61.3%)	356 (45.0%)	<0.001
	Diabetes (n)	456 (28.0%)	200 (25.3%)	0.169
	Atrial fibrillation (n)	491 (30.2%)	346 (43.7%)	<0.001
	Stroke (n)	201 (12.4%)	92 (11.6%)	0.656
	Chronic obstructive pulmonary disease (n)	115 (7.07%)	63 (7.96%)	0.478
	Thyroid disease (n)	11 (0.68%)	17 (2.15%)	0.003
	Chronic kidney disease (n)	190 (11.7%)	127 (16.1%)	0.003
Echocardiogram				
	Ejection fraction (%)	50.8 (36.0–61.3)	33.0 (25.0–46.0)	<0.001
	Interventricular septum thickness in diastolic phase (cm)	1.00 (0.90–1.09)	0.95 (0.85–1.05)	<0.001
	Left atrium dimension (cm)	4.05 (3.62–4.54)	4.68 (4.24–5.08)	<0.001
	Left ventricular end-diastolic volume (mL)	130 (102–171)	162 (126–212)	<0.001
	Left ventricular internal diameter in diastolic phase (cm)	5.17 (4.60–5.87)	6.08 (5.30–6.72)	<0.001
	Left ventricular posterior wall thickness in diastolic phase (cm)	0.99 (0.91–1.08)	0.95 (0.85–1.05)	<0.001
	Tricuspid reugrgitation	241 (14.8%)	370 (46.8%)	<0.001

All values are presented as the means ± SD or n (%) or as the median (interquartile range). n, number of individuals; MR, mitral regurgitation.

**Table 3.** **Baseline clinical characteristics of MR vs. non-MR**.

		non-MR	MR	*p*
		(n = 1627)	(n = 791)	
Laboratory result				
	BNP (pg/mL)	206 (64.6–524)	574 (268–1346)	<0.001
	NT-proBNP (pg/mL)	1458 (488–3828)	3478 (1522–7178)	<0.001
	CRP (mg/L)	5.00 (5.00–11.6)	5.70 (5.00–14.0)	0.002
	Cr (umol/L)	77.0 (64.0–98.0)	83.0 (68.0–110)	<0.001
	Hb (g/L)	131 (118–145)	129 (115–142)	0.006
	ALT (u/L)	30.0 (23.0–40.2)	31.0 (24.0–44.0)	0.001
	HbA1c (%)	6.20 (5.80–6.90)	6.20 (5.80–6.80)	0.410
	FFA (umol/L)	451 (315–631)	528 (356–726)	<0.001
	BHB (mmol/L)	0.06 (0.04–0.10)	0.08 (0.05–0.19)	<0.001
	Glu (mmol/L)	6.30 (5.24–8.06)	6.31 (5.16–7.86)	0.239
	TG (mmol/L)	1.16 (0.88–1.64)	1.02 (0.77–1.37)	<0.001
	HDL (mmol/L)	1.12 (0.96–1.32)	1.13 (0.92–1.32)	0.364
	LDL (mmol/L)	1.85 (1.39–2.42)	1.88 (1.37–2.38)	0.784
Medication				
	ACEI	680 (41.8%)	391 (49.4%)	<0.001
	ARB	581 (35.7%)	234 (29.6%)	0.003
	ARNi	138 (8.48%)	136 (17.2%)	<0.001
	β-blocker	1273 (78.2%)	678 (85.7%)	<0.001
	Spironolactone	974 (59.9%)	675 (85.3%)	<0.001
	Diuretic	1079 (66.3%)	714 (90.3%)	<0.001
	Digoxin	296 (18.2%)	316 (39.9%)	<0.001
	Amiodarone	207 (12.7%)	164 (20.7%)	<0.001
	Anti-platelet	204 (12.5%)	48 (6.07%)	<0.001
	Statin	1361 (83.7%)	567 (71.7%)	<0.001
	Insulin	273 (16.8%)	127 (16.1%)	0.696
	Metformin	143 (8.79%)	56 (7.08%)	0.175
	Trimetazidine	515 (31.7%)	313 (39.6%)	<0.001
Intervention				
	ICD	30 (1.84%)	53 (6.70%)	<0.001
	CRT	25 (1.54%)	33 (4.17%)	<0.001
	PCI	375 (23.0%)	113 (14.3%)	<0.001
	Ablation	90 (5.53%)	49 (6.19%)	0.573

All values are presented as the means ± SD or n (%) or as the median (interquartile range). n, number of individuals; BNP, brain natriuretic peptide; NT-proBNP, N-terminal pro-B type natriuretic peptide; CRP, C-reactive protein; Cr, creatine; Hb, hemoglobin; ALT, alanine aminotransferase; HbA1c, glycated hemoglobin; FFA, free fatty acid; BHB, β-hydroxybutyrate; Glu, glucose; TG, triglycerides; HDL, high density lipoprotein; LDL, low density lipoprotein; ACEI, angiotensin converting enzyme inhibitor; ARNi, angiotensin receptor neprilysin inhibitor; ARB, angiotensin receptor blocker; ICD, implantable cardioverter defibrillator; CRT, cardiac resynchronization therapy; PCI, percutaneous coronary intervention.

### 3.3 Associated Factors Analysis

**Fig. 2.**
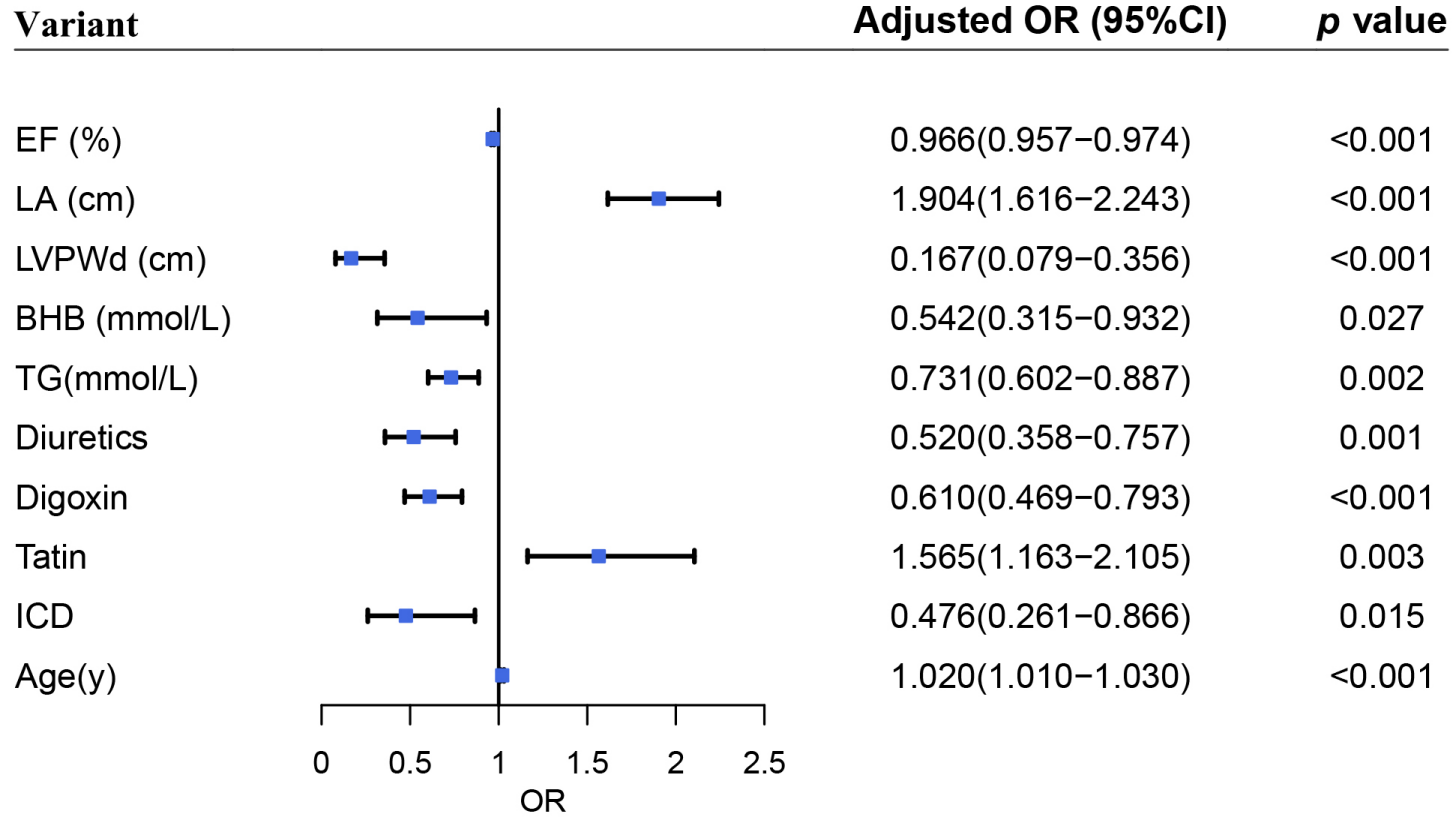
**Forest plot on associated factors of functional mitral regurgitation**.

Univariable analysis data was displayed in **Supplementary Table 1**. Stepwise multivariable logistic regression using likelihood ratio method was utilized to identify the associated factors of MR. The result showed that EF (OR 0.966, 95% confidence interval (CI) [0.957, 0.974], *p <* 0.001), LVPWd (OR 0.167, 95% CI [0.079, 0.356], *p <* 0.001), BHB (OR 0.542, 95% CI [0.315, 0.932], *p *= 0.027), TG (OR 0.731, 95% CI [0.602, 0.887], *p *= 0.002), diuretics (OR 0.520, 95% CI [0.358, 0.757], *p *= 0.001), digoxin (OR 0.610, 95% CI [0.469, 0.793], *p <* 0.001), and ICD (OR 0.476, 95% CI [0.261, 0.866], *p *= 0.015) associated with less MR. The result showed that age (OR 1.020, 95% CI [1.010, 1.030],* p <* 0.001), statin (OR 1.565, 95% CI [1.163, 2.105], *p *= 0.003), LA dimension (OR 1.904, 95% CI [1.616, 2.243], *p *= 0.000) associated with more MR (Fig. 2). Due to the significant difference between HFpEF, HFmEF and HFrEF groups displayed before (Table 1), we classified HF patients into three groups according to EF, and conducted logistic regression.

### 3.4 Associated Factors of MR in HFpEF 

For patients with HFpEF, four factors associated with MR incuding EF (OR 0.954 (0.928–0.981), *p *= 0.001), LVPWd (OR 0.274 (0.081–0.932), *p *= 0.038), LA dimension (OR 2.049 (1.631–2.576), *p <* 0.001) and age (OR 1.024 (1.007–1.041), *p *= 0.007) after adjusting for confounders (Table 4).

**Table 4.** **Factors associated with MR for heart failure patients classified by ejection fraction**.

		Model 1		Model 2	
		Adjusted OR (95% CI)	*p*	Adjusted OR (95% CI)	*p*
HFpEF					
	EF (%)	0.955 (0.927–0.983)	0.002	0.954 (0.928–0.981)	0.001
	LA dimension (cm)	1.923 (1.527–2.423)	<0.001	2.049 (1.631–2.576)	<0.001
	LVPWd (cm)	0.260 (0.073–0.928)	0.038	0.274 (0.081–0.932)	0.038
	Diuretic	0.453 (0.284–0.723)	0.001	0.549 (0.348–0.867)	0.100
	Statin	1.736 (1.063–2.836)	0.028	1.368 (0.805–2.322)	0.246
	Age	1.022 (1.005–1.040)	0.013	1.024 (1.007–1.041)	0.007
HFmrEF					
	LA dimension (cm)	1.904 (1.355–2.675)	<0.001	2.009 (1.427–2.829)	<0.001
	TG (mmol/L)	0.562 (0.365–0.865)	0.009	0.552 (0.359–0.849)	0.007
	Digoxin	0.315 (0.183–0.540)	<0.001	2.836 (1.624–4.951)	<0.001
HFrEF					
	EF (%)	0.969 (0.949–0.991)	0.005	0.969 (0.949–0.990)	0.004
	LA dimension (cm)	2.222 (1.776–2.779)	<0.001	2.289 (1.821–2.878)	<0.001
	LVPWd (cm)	0.145 (0.060–0.352)	<0.001	0.161 (0.067–0.387)	<0.001
	TG (mmol/L)	0.744 (0.594–0.933)	0.010	0.746 (0.595–0.936)	0.011
	Diuretic	0.518 (0.309–0.869)	0.013	0.559 (0.334–0.934)	0.026
	Statin	1.443 (1.033–2.015)	0.031	1.312 (0.917–1.878)	0.138
	ICD	0.483 (0.272–0.857)	0.013	1.898 (1.074–3.354)	0.027
	Age	1.017 (1.006–1.029)	0.003	1.016 (1.004–1.027)	0.009

Model 1 multivariable logistic regression model; Model 2 adjusted for sex, CAD and DM. FMR, functional mitral regurgitation; OR, odds ratio; CI, confidence interval; EF, ejection fraction; LA, left atrium; LVPWd, left ventricular posterior wall thickness in diastolic phase; TG, triglycerides; ICD, implantable cardioverter defibrillator.

### 3.5 Associated Factors of MR in HFmEF 

For patients with HFmrEF, three factors associated with MR including LA dimension (OR 2.009 (1.427–2.829), *p <* 0.001) and digoxin (OR 2.836 (1.624–4.951), *p <* 0.001) after adjusting for confounders (Table 4). Surprisingly, triglycerides (TG) (OR 0.552 (0.359–0.849), *p *= 0.007) seemed to be related to MR in this subgroup.

### 3.6 Associated Factors of MR in HFrEF 

For patients with HFrEF, seven factors associated with MR: EF (OR 0.969 (0.949–0.990), *p *= 0.004), LVPWd (OR 0.161 (0.067–0.387), *p <* 0.001), LA dimension (OR 2.289 (1.821–2.878), *p <* 0.001), age (OR 1.016 (1.004–1.027)), *p *= 0.009) and diuretics (OR 0.559 (0.334–0.934), *p *= 0.026) after adjusting for confounders (Table 4). Surprisingly, TG (OR 0.746 (0.595–0.936), *p *= 0.011) and ICD (OR 1.898 (1.074–3.354), *p *= 0.027) seemed to be related to MR in this subgroup.

## 4. Discussion

The prevalence and characteristics of MR in HF patients (including HFpEF) were determined in this study using data from a Chinese hospital. To begin, a significant proportion of HF patients in our study had MR, particularly the HFrEF patients. Due to the high prevalence of HF in China, a significant proportion of MR patients have a poor prognosis and may benefit from mitral valve repair, as clinical trials continue [20, 21]. Second, MR is associated with deteriorated cardiac function, including atrial and ventricle abnormalities. It is worth noting that the LA dimension appeared to be critical in the progression of HF and MR. Third, older age was associated with increased MR. Finally, it appeared as though TG, diuretics, digoxin, and ICD were associated with decreased MR.

Our cross-sectional study found a high prevalence of MR in HF patients (32.71%) and an increasing ratio in HFrEF patients (49.70%). Similar findings have been reported. A previous study showed 44.5% MR in acute HF patients (EF <50%) and 27.5% MR in acute HF patients (EF >50%) in the United States [4]. However, another study reported a lower proportion of MR (6.1%) in Japan [5]. This discrepancy could be explained by differences in medical therapy strategies, which may have influenced the progression of MR. Both guideline-directed medicine therapy (GDMT) and transcatheter mitral valve repair have been shown to slow the progression of MR. A randomized trial of transcatheter mitral valve repair showed that 10.61% of HF patients in the control group receiving recommended medication therapy for one year had a recovered MR in the prospective study; by contrast, the device group had a higher proportion (37.42%) [22]. Similar results were observed in Chinese patients [21]. We then examined the characteristics and risk factors for MR in greater detail. MR patients were characterized by elder age, increased heart rate, and lower BMI, consistent with previous studies [4, 5]. Conclusively, MR remained a high prevalence in HF and appeared to be a dynamic condition associated with HF progression. Thus, MR might serve as a promising therapeutic target for HF patients.

A significant finding was that MR was associated with a deteriorated cardiac function (EF), a thinner wall thickness (LVPWd), and a larger LA chamber (LA dimension), as determined by the etiology. Previous studies have demonstrated that a proportion of AF patients exhibit atrial MR (most commonly in HFpEF patients) characterized by LA and mitral annular enlargement [23, 24]. In the meanwhile, conventional MR was characterized by LV enlargement and papillary muscle dysfunction. We classified HF patients by ejection fraction to investigate the clinical characteristics of MR in different HF subtypes. LA dimension appeared to be associated with MR in patients with HFpEF (OR 2.049 (1.631–2.576),* p <* 0.001) and HFrEF (OR 2.289 (1.821–2.878), *p <* 0.001). Of note, in patients with HFmrEF, only LA dimension (OR 1.904, *p <* 0.001) seemed to independently associate with MR, while the logistic regression model excluded EF and LVPWd. This finding might be explained by the high prevalence of AF (40.5%) in the HFmrEF group. It suggested that atrial MR might have an effect on this type of HF patient. The majority of atrial MR studies have been conducted on patients with HFpEF [25, 26]; Our findings suggested that atrial etiology may be involved in both HFmrEF and HFrEF.

While LA dimension was found to be significantly associated with MR, there was a significant interaction between LA dimension and AF. AF, HF, and MR appeared to construct a vicious circle, which could be broken to delay the progression of HF. On the one hand, worsened MR seemed to aggravate the burden of left atrium, resulting in the enlargement and dysfunction of LA, which eventually developed into AF [27]. On the other hand, recent studies indicated that atrial MR seemed to be a novel subtype characterized by significant dilatation of mitral annulus and LA [23, 28, 29]. A randomized clinical trial published in 2018 enrolled 363 patients with HF (EF <35%) and AF. After a median follow-up of 37.8 months, the primary composite endpoint (re-hospitalized for worsening heart failure or died from any cause) occurred in significantly fewer patients in the ablation group than in the medical therapy group (51 patients [28.5%] vs. 82 patients [44.6%]; hazard ratio, 0.62; 95% confidence interval (CI) [0.43–0.87]; *p *= 0.007) [30]. Additionally, Park *et al*. [31] reported that after the mitral loop cerclage procedure, patients with MR and persistent atrial fibrillation spontaneously reverted to sinus rhythm with reduced MR. However, evidence from a large-scale clinical trial is needed for the broader promotion of this hypothesis. Conclusively, MR was a promising therapeutic target for HF and AF patients.

Demographic differences in MR, including sex, age, and race, were reported. Elderly patients were more likely to associate with progressive HF and worsen MR [11, 12]. The white and the female were more likely to have moderate to severe MR [8]. We found that BHB (OR 0.542, 95% CI [0.315–0.932], *p *= 0.002) and TG (OR 0.731, 95% CI [0.602–0.887], *p *= 0.002) might associate with MR. BHB, a ketone body, served as an energy supply for the heart, especially during heart failure [32]. Several clinical trials tested the external supplement of BHB and received positive results, which seemed to strengthen the cardiac function and possibly ameliorate MR condition [33, 34]. We found that a lower level of TG was associated with MR. However, a previous study pointed out that a high serum level of TG is associated with the later development of heart failure [35]. BHB and TG might serve as potential markers of MR though these applications should be investigated in further studies.

The use of diuretics and digoxin included in the guideline-directed medical therapy of HF was associated with less MR [16, 36]. Clinical trials failed to demonstrate the benefit of statins in patients with HF [37]; furthermore, our research found that the use of statins associated with more MR, which indicated that statins might be deleterious for patients with MR. However, subgroup analysis seemed not to support this hypothesis. The implantation of ICD seemed to be associated with MR. Previous studies have demonstrated that CRT reduces the volume of mitral regurgitation. ICD was recommended for patients with asymptomatic LV systolic dysfunction (LVEF <30%) of the ischemic origin or asymptomatic non-ischemic dilated cardiomyopathy (LVEF <30%) who received GDMT [16]. These patients were vulnerable to MR, according to our results and studies before [4, 38]. However, the logistic model demonstrated that ICD was associated with less MR. Conclusively, HF treatment strategy influences MR.

### Limitation

This was a chart review study, and the authors are aware of the limitations. Firstly, this was a retrospective study conducted in a single center. To minimize biases, we adopted standardized data extraction; multivariable logistic regression was adopted in order to adjust for well-known confounders. We further classified HF into three groups due to the significantly different etiology between them and adjusted for known confounders. Secondly, it was a chart review study; echocardiogram diagnosis and parameters were acquired from medical records at diagnosis instead of post-analysis from echocardiogram image. As such, our results should be interpreted with caution. Notably, the echocardiologists in our center applied parameters including effective regurgitation orifice area and regurgitation volume when diagnosing severe regurgitation; however, these parameters were not available in the medical records. Thirdly, there were missing values in the original data set; we excluded the records when missing values existed instead of replacing them.

## 5. Conclusions

Our research showed that in a large Chinese hospital, MR had a high prevalence in patients with HF. Worsen cardiac structure (LA dimension and LVPWd) and function (EF), age, and medical treatment strategy played important roles in MR. Among them, LA dimension is of great importance in subtypes of HF. These findings described the characteristics and etiology of MR in the clinical context.

## Supplementary Material

Supplementary material associated with this article can be found, in the online version, at https://doi.org/10.31083/j.rcm2307235.
