# Supplementary material for: The Prevalence and Characteristics of Mitral Regurgitation in Heart Failure: A Chart Review Study
Source: Rev Cardiovasc Med. 2022 Jun 24;23(7):235. doi: 10.31083/j.rcm2307235 (PMC11266808; doi:10.31083/j.rcm2307235)
Supplement: Supplementary file 1 [file 2153-8174-23-7-235-s1.docx]

Supplementary Table 1. Result of univariate logistic regression analysis.

| Variable | OR（95% CI） | *p* | variable | OR（95% CI） | *p* |
| --- | --- | --- | --- | --- | --- |
| EF | 0.950 (0.956–0.944) | 0.000 | Thyroid disease | 3.227 (7.131–1.521) | 0.003 |
| IVSd | 0.223 (0.357–0.137) | 0.000 | CKD | 1.447 (1.842–1.133) | 0.003 |
| LA dimension | 2.690 (3.063–2.370) | 0.000 | ACEI | 1.361 (1.615–1.148) | 0.000 |
| LVEDV | 1.008 (1.010–1.006) | 0.000 | ARB | 0.756 (0.908–0.629) | 0.003 |
| LVIDd | 2.098 (2.305–1.915) | 0.000 | ARNi | 2.240 (2.889–1.737) | 0.000 |
| LVPWd | 0.147 (0.255–0.083) | 0.000 | β-blocker | 1.668 (2.110–1.328) | 0.000 |
| LVPWs | 0.136 (0.199–0.092) | 0.000 | Spironolactone | 3.901 (4.882–3.140) | 0.000 |
| BNP | 1.000 (1.001–1.000) | 0.000 | Diuretic | 4.709 (6.127–3.665) | 0.000 |
| C-reactive protein | 1.001 (1.004–0.997) | 0.624 | Digoxin | 2.991 (3.619–2.474) | 0.000 |
| BHB | 1.674 (2.511–1.125) | 0.011 | Amiodarone | 1.794 (2.247–1.431) | 0.000 |
| LDL | 0.954 (1.083–0.839) | 0.472 | Anti-platelet | 0.451 (0.619–0.322) | 0.000 |
| NT-proBNP | 1.000 (1.000–1.000) | 0.000 | Statin | 0.495 (0.606–0.404) | 0.000 |
| FFA | 1.001 (1.001–1.000) | 0.000 | Insulin | 0.949 (1.192–0.752) | 0.653 |
| TG | 0.617 (0.714–0.529) | 0.000 | Metformin | 0.791 (1.084–0.569) | 0.152 |
| HbA1c | 0.967 (1.043–0.895) | 0.393 | Trimetazidine | 1.414 (1.687–1.185) | 0.000 |
| Cr | 1.002 (1.003–1.001) | 0.006 | Body mass index | 0.920 (0.942–0.898) | 0.000 |
| Glu | 0.976 (1.006–0.947) | 0.119 | ICD | 3.823 (6.099–2.440) | 0.000 |
| Hb | 0.995 (0.999–0.991) | 0.012 | CRT | 2.790 (4.767–1.653) | 0.000 |
| ALT | 1.000 (1.001–1.000) | 0.095 | PCI | 0.556 (0.698–0.441) | 0.000 |
| HDL | 0.836 (1.165–0.597) | 0.292 | Ablation | 1.128 (1.607–0.783) | 0.511 |
| Hypertension | 0.663 (0.787–0.559) | 0.000 | Age | 1.007 (1.014–1.001) | 0.029 |
| Coronary artery disease | 0.517 (0.614–0.435) | 0.000 | Sex (female) | 1.119 (1.341–0.933) | 0.222 |
| Diabetes | 0.869 (1.053–0.715) | 0.155 | Systolic blood pressure | 0.988 (0.993–0.983) | 0.000 |
| Atrial fibrillation | 1.799 (2.145–1.509) | 0.000 | Diastolic blood pressure | 0.994 (1.001–0.986) | 0.106 |
| Stroke | 0.934 (1.211–0.715) | 0.609 | Heart rate | 1.006 (1.012–1.000) | 0.048 |
| COPD | 1.138 (1.561–0.823) | 0.429 |  |  |  |

FMR, functional mitral regurgitation; OR, odds ratio; CI, confidence interval; EF, ejection fraction; LA, left atrium; LVPWd, left ventricular posterior wall thickness in diastolic phase; LVPWs, left ventricular posterior wall thickness in systolic phase; LVIDd, left ventricular interval diameter in diastolic phase; LVEDV, left ventricular end diastolic volume; BNP, brain natriuretic peptide; NT-proBNP, N-terminal pro-B type natriuretic peptide; Cr, creatine; Hb, hemoglobin; ALT, alanine aminotransferase; HbA1c, glycated hemoglobin; FFA, free fatty acid; BHB, β–hydroxybutyrate; Glu, glucose; TG, triglycerides; HDL, high density lipoprotein; LDL, low density lipoprotein; ACEI, angiotensin converting enzyme inhibitor; ARNi, angiotensin receptor neprilysin inhibitor; ARB, angiotensin receptor blocker; ICD, implantable cardioverter defibrillator; CRT, cardiac resynchronization therapy; PCI, percutaneous coronary intervention; COPD, chronic obstructive pulmonary disease.
